# Optimization and Implication of Adipose-Derived Stem Cells in Craniofacial Bone Regeneration and Repair

**DOI:** 10.3390/bioengineering11111100

**Published:** 2024-10-31

**Authors:** Cong Gu, Qinghuang Tang, Liwen Li, YiPing Chen

**Affiliations:** 1 Department of Cell and Molecular Biology, Tulane University, New Orleans, LA 70118, USA; qinghuan@buffalo.edu (Q.T.); lli88@buffalo.edu (L.L.); ychen@tulane.edu (Y.C.); 2 Department of Oral Biology, School of Dental Medicine, University at Buffalo, Buffalo, NY 14214, USA; 3 Department of Biological Sciences, University at Buffalo, Buffalo, NY 14260, USA

**Keywords:** adipose-derived stem cells, ADSCs, craniofacial bone regeneration

## Abstract

Adipose-derived stem cells (ADSCs) have emerged as a promising resource for craniofacial bone regeneration due to their high abundance and easy accessibility, significant osteogenic potential, versatile applications, and potential for personalized medicine, which underscore their importance in this field. This article reviews the current progress of preclinical studies that describe the careful selection of specific ADSC subpopulations, key signaling pathways involved, and usage of various strategies to enhance the osteogenic potential of ADSCs. Additionally, clinical case reports regarding the application of ADSCs in the repair of calvarial defects, cranio-maxillofacial defects, and alveolar bone defects are also discussed.

## 1. Introduction

Craniofacial defects are a group of abnormalities that damage the structure, function, and aesthetic appearance of the head and face of patients. These defects can vary widely in severity, type, and causes, such as cleft lip and palate, craniosynostosis, positional plagiocephaly, apert syndrome, and craniofacial trauma, which are caused congenitally by genetic mutations or caused by environmental factors or accidents [1,2,3,4,5,6]. Conventional treatments for craniofacial regeneration encompass various approaches that aim to repair or restore the skull and face to correct defects or heal injuries, which usually involve surgical techniques and tissue engineering [7,8]. However, the final outcomes of traditional methods can sometimes be unsatisfactory and less efficacious due to the limited tissue availability for autologous grafts, high immunogenicity with allografts, inability to fully restore the complex sensory and motor functions of craniofacial structures, and inflammation after surgical procedure [9]. Stem cells are undifferentiated cells that have the potential to self-renew and differentiate into other specialized cell types, which are essential for tissue repair [10]. Instead of the limited supply of tissues used in conventional strategies, stem cells can be isolated from various self-sources such as bone marrow, adipose tissue, and dental pulp and can even be generated by reprogramming adult cells to a pluripotent state [11]. Additionally, isolated tissue-specific stem cells maintain the potential to differentiate into the multiple cell types that are needed for craniofacial tissue repair. Therefore, stem cell therapy has emerged as a promising approach that minimizes the disadvantages of conventional strategies and offers potential solutions for repairing and reconstructing complex craniofacial structures. Among all the different types of stem cells, adipose-derived stem cells (ADSCs) are currently considered the most promising candidate for the treatment of cranio-maxillofacial bone defects [12]. Thus, the aim of this review is to investigate the tailored selection of ADSC subpopulations for craniofacial bone reconstruction, the underlying molecular mechanisms of ADSC osteogenic differentiation, different strategies to enhance bone formation, and up-to-date clinical case reports of ADSCs in craniofacial bone regeneration.

## 2. ADSC Properties

ADSCs are a type of mesenchymal stem cell (MSC) that display a typical fibroblast-like morphology [13]. ADSCs are abundant and can be easily harvested from various subcutaneous adipose tissues such as inguinal and abdominal fat depots [14,15] or from visceral sites [16]. For the purpose of the clinical application of human ADSCs, a common liposuction procedure is usually applied, characterized by a hollow blunt-tipped cannula infused into the subcutaneous adipose tissue to suck the fat [17,18], followed by enzymatic digestion to break down the extracellular matrix in the fat tissue and subsequent centrifugation to enrich the population of ADSCs [19]. Notably, the age of the donor and multiple passages have a less pronounced effect on the proliferation and osteogenic differentiation capabilities of isolated ADSCs compared to bone marrow-derived stem cells (BMSCs) [20]. Furthermore, it is widely accepted that ADSCs maintain the multipotency to differentiate into a variety of cell types such as adipocytes, osteocytes, chondrocytes, muscle cells, and neural cells in vitro [21,22,23], with the treatment of corresponding lineage-specific induction factors, though ADSCs are of mesodermal origin [24]. Gou et al. compared the osteogenic potential of MSCs derived from different tissue sources, with a focus on identifying the best candidate for bone tissue engineering [25]. They reported that ADSCs exhibit the strongest osteogenic and adipogenic potential in vitro and in vivo, particularly when stimulated by bone morphogenetic protein-9 (BMP9), as compared to that of mouse embryonic fibroblasts, BMSCs, and calvarial mesenchymal progenitors, suggesting that ADSCs are a superior cell source for bone tissue engineering applications [25]. The versatility in differentiation makes ADSCs a promising cell source for repairing complex craniofacial defects, especially for bone repair, offering potential improvements over traditional reconstructive methods.

## 3. Selection of ADSC Subpopulations for Bone Repair

**Figure 1:**
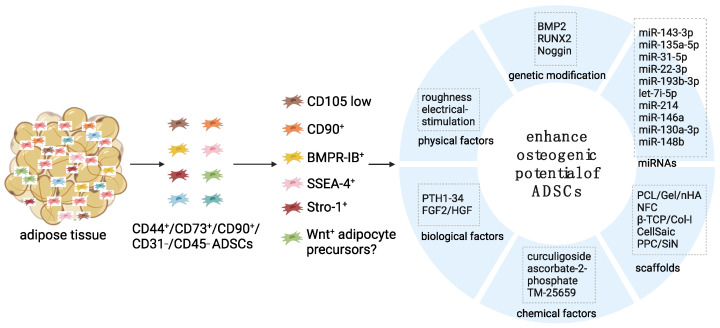
Strategies to enhance the osteogenic potential of ADSCs. Created in BioRender. Gu, C. (2024) https://BioRender.com/s91j309. accessed on 23 October 2024.

The stromal vascular fraction of adipose tissue is heterogeneous. It comprises a diverse array of cell types, including ADSCs [26], endothelial cells [27,28], smooth muscle cells [29], fibroblasts [30], pericytes [31], macrophages [32], lymphocytes [33], and various progenitors [34,35,36]. To isolate the ADSC subpopulations, positive markers such as CD90, CD44, CD29, CD105, CD13, CD34, CD73, CD166, CD10, CD49e, and CD59 and negative markers such as CD31, CD45, CD14, CD11b, CD34, CD19, CD56, and CD146 are generally used [16,37,38,39]. Notably, ADSCs also contain heterogeneous subpopulations with different differentiation potential. Wu and colleagues discovered that the CD146-positive ADSC subtype plays a better role in reducing knee osteoarthritis pain and facilitates cartilage repair in rats [40]. Additionally, another study reported that c-Kit-positive ADSCs exhibit higher adipogenic efficiency and show an enhanced self-renewal ability [41]. A more recent study described the residing of six different clusters of ADSCs in the upper body abdominal and/or lower body gluteofemoral adipose tissues of humans, three of which exhibited stronger cholesterol/fatty acid storage and one of which showed characteristics like smooth muscles cells and weaker differentiation capacity in vitro [42]. Therefore, identifying specific subpopulations of ADSCs that possess enhanced osteogenic potential is crucial for the optimization of stem cell-based therapies of bone regeneration (Figure 1).

By utilizing microfluidic-based single-cell transcriptional analysis, Levi et al. found that the low expression of endoglin (CD105) is highly correlated with enhanced osteogenic gene expression profiles in a subpopulation of ADSCs derived from human subcutaneous adipose tissue [43]. Additionally, the study revealed that the reduced expression of CD105 can lead to decreased transforming growth factor beta 1 (TGF-β1)/SMAD family member 2 (Smad2) signaling, which is known to inhibit osteogenesis, suggesting that targeting CD105 can improve the efficacy of ADSCs in bone regeneration therapies by enhancing their osteogenic differentiation [43]. Notably, a follow-up study by the same group further identified a second cell surface receptor Thy-1 (CD90) that marks another subpopulation of ADSCs that exhibits significantly higher alkaline phosphatase (ALP) activity, greater extracellular matrix mineralization, and the upregulation of osteogenic genes like runt-related transcription factor 2 (*RUNX2*), osteopontin (OPN), and osteocalcin (*OCN*) as compared to CD105low cells in vitro [44]. In vivo experiments using a calvarial defect model in mice also confirmed that CD90+ cells lead to more robust bone regeneration, indicating that CD90 is a more effective marker for identifying ADSC subtypes with stronger osteogenic differentiation potential for bone tissue engineering than CD105 [44].

McArdle et al. isolated a bone morphogenetic protein receptor type-IB (BMPR-IB)-positive ADSC subpopulation using magnetic cell sorting and demonstrated that these cells have a significantly higher osteogenic potential compared to that of BMPR-IB-negative or unsorted ADSCs [45]. These BMPR-IB-positive cells showed greater ALP activity in cell cultures starting at day 7 and enhanced extracellular matrix mineralization starting at day 14. Additionally, the BMPR-IB-positive ADSCs significantly improved bone regeneration in a critical-sized calvarial defect model in mice, suggesting that BMPR-IB-positive ADSCs are a promising cell population for bone tissue engineering [45]. Moreover, a subpopulation of the stromal vascular fractions from human adipose tissue, characterized by the expression of the pluripotency-associated marker SSEA-4, was shown to possess a superior ability to differentiate into the osteogenic lineage, as compared to other populations of human ADSCs [46]. The treatment of an SSEA-4+ subpopulation of ADSCs with silicate nanoplatelets (sNPs) at a concentration below 100 mg/mL appeared cytocompatible but promoted osteogenic differentiation by enhancing the expression of key osteogenic markers, such as *RUNX2*, *OPN*, and *OCN* [47]. Furthermore, the addition of sNPs led to increased ALP activity and robust matrix mineralization, covering more than 90% of the culture surface [47]. The findings suggest that combining SSEA-4+ ADSCs with sNPs holds significant potential for bone tissue engineering applications. It was also reported that different subpopulations of human ADSCs exhibited varying levels of chondrogenic and osteogenic differentiation potential [48]. Notably, subpopulations isolated using anti-STRO-1 antibodies exhibited the highest osteogenic potential, while others, such as those isolated with anti-CD29 and anti-CD73 antibodies, showed greater chondrogenic potential [48]. These findings underscore the importance of selecting specific ADSC subpopulations for targeted applications in bone and cartilage tissue engineering.

Previously, we discovered a novel population of adipocytes with active intracellular Wnt/β-catenin signaling, named Wnt^+^ adipocytes [49]. These adipocytes are distinct from conventional adipocytes in which Wnt/β-catenin signaling is not required and instead maintains preadipocytes in an undifferentiated state [50]. Single-cell RNA-seq, single-cell ATAC-seq, and Seahorse assay (oxygen consumption rate) results indicated that Wnt^+^ adipocytes exhibit higher metabolic and thermogenic characters compared to those of Wnt^-^ adipocytes. Considering that higher mitochondrial respiration and oxidative phosphorylation are closely correlated with a higher osteogenic potential in stem cells [51,52,53], and the multipotency of ADSCs, we hypothesized that Wnt^+^ adipocyte precursors can also differentiate into osteocytes with enhanced osteogenic potential. We found that the precursors of either Wnt^+^ adipocytes or Wnt^-^ adipocytes indeed differentiated into osteocytes after osteogenic induction, but those of Wnt^+^ adipocytes exhibited much faster and stronger osteogenic differentiation capacity, implicating a potential application of such Wnt^+^ adipocyte precursors in future craniofacial bone regeneration.

## 4. Key Signaling Pathways Involved in the Regulation of Osteogenic Potential of ADSCs

The osteogenic differentiation of ADSCs is governed by the integration of several key signaling pathways that involve the interplay of various molecular mechanisms, transcription factors, and signaling cascades. Understanding the underlying mechanisms allows for the modulation of these pathways through various interventions, such as genetic modifications and the application of growth factors and/or small molecules, to enhance the differentiation of ADSCs into osteoblasts. 

### 4.1. TGF-β/BMP Signaling

TGF-β/BMP signaling plays a crucial role in regulating bone formation and remodeling and in maintaining the balance between osteoblasts and osteoclasts [54,55,56,57]. Both TGF-β and BMP signaling operate through two distinct mechanisms, the canonical Smad-dependent pathway and the non-canonical Smad-independent TAK1 pathway, which include signaling cascades such as TGF-β/BMP ligands, receptors, and Smad proteins or the p38 mitogen-activated protein kinase (MAPK) pathway, respectively [58,59]. Upon TGF-β/BMP activation, both the Smad and p38 MAPK pathways converge on the *RUNX2* gene, a key regulator of mesenchymal precursor cell differentiation [55]. Liu et al. reported that the treatment of rat ADSCs with osteogenic medium containing TGF-β1 and *BMP2* significantly increases the expression of *Smad1*, *Smad5*, and *Smad8* and key osteogenic markers like *RUNX2*, collagen type I (*Col-I*), and *OPN*, suggesting that TGF-β/BMP signaling is essential for the osteoblast differentiation of ADSCs [60]. Consistently, another study described that an optimal concentration of 80 µg/mL of monodispersed bioactive glass nanoparticles can be efficiently internalized by ADSCs and activate the TGF-β/Smad3 signaling pathway to enhance osteogenic differentiation, as evidenced by the increased ALP activity, calcium deposition, and expression of osteogenic markers [61]. These findings suggest that targeting TGF-β/BMP signaling in ADSCs would benefit bone tissue engineering.

### 4.2. Notch Signaling

Notch signaling is an evolutionarily conserved intercellular pathway that regulates cell proliferation, cell fate, and differentiation [62,63]. It is widely accepted that Notch proteins are key regulators in osteogenesis [64,65]. Nevertheless, the precise role of Notch in regulating the osteogenic potential of ADSCs remains controversial. Several groups reported that Notch signaling inhibits osteogenesis [66,67], while others found that the activation of Notch signaling promotes the differentiation of osteocytes [68,69]. Lough et al. reported that inhibiting Notch signaling impedes ADSC proliferation and osteogenic differentiation, but these effects can be reversed by introducing the constitutively active Notch constructs, Notch-1 intracellular domain, suggesting a potential “on/off switch” for controlling the bone formation of ADSCs [70]. Notably, the constitutively active Notch signaling achieved via genetic manipulation may not represent the natural physiological conditions. Therefore, understanding the precise regulatory mechanism of Notch signaling in ADSCs is required for craniofacial bone tissue engineering to speed up clinical applications.

### 4.3. Hedgehog Signaling

The hedgehog signaling pathway is a highly conserved pathway that is essential for embryonic development, tissue homeostasis, and regeneration [71]. In mammals, three hedgehog ligands have been discovered: Sonic hedgehog, Indian hedgehog, and Desert hedgehog [72]. The binding of hedgehog ligands to their transmembrane receptor Patched1 leads to the activation of the transmembrane protein smoothened and the subsequent activation of downstream Gli transcription factors, which plays a crucial role in promoting osteogenesis [73,74]. Hedgehog signaling, particularly triggered by Sonic hedgehog that is derived from human ADSCs, was identified crucially in promoting the osteogenic differentiation of mouse calvarial osteoblasts [75]. In consistence with this, cyclopamine, a hedgehog pathway antagonist, significantly inhibited bone formation. In vivo studies using a mouse calvarial defect model further demonstrated that ADSCs enhance bone healing through the activation of the hedgehog signaling pathway [75]. Thus, hedgehog signaling appears to play a critical role in the process of ADSC-mediated bone repair.

### 4.4. Wnt Signaling

The Wnt signaling pathway is an ancient pathway that mediates crucial aspects like cell fate determination, embryogenesis, and organogenesis [76]. The activation of Wnt signaling is initiated by the binding of Wnt ligands to Frizzled receptors, which leads to the formation of a cell surface complex with G-protein-coupled receptors and receptor-related proteins and the subsequent activation of four distinct signaling pathways depending on the cell-specific contexts [76,77,78,79]. The canonical Wnt signaling or Wnt/β-catenin has been reported to be involved in the osteoblast differentiation of mesenchymal cells and skeletal development via the axis of Wnt/β-catenin/TCF1/*RUNX2* [80]. Li et al. studied the effects of Salidroside, a natural compound derived from Rhodiola rosea L, on the osteogenic differentiation of ADSCs [81]. They found that Salidroside significantly enhances the osteogenic capacity of ADSCs by promoting cell viability, increasing ALP activity, and enhancing calcium deposition. RNA sequencing further revealed that Salidroside induces the upregulation of 345 genes and downregulation of 198 genes, with many of these involved in the Wnt/β-catenin signaling pathway [81]. Additionally, the silencing of β-catenin partially reversed the osteogenic effects of Salidroside, confirming the critical role of the Wnt/β-catenin pathway in the osteogenic differentiation of ADSCs [81].

### 4.5. ERK1/2 Signaling

The extracellular signal-regulated kinase 1/2 (*ERK1/2*) signaling pathway plays a central role in signal transduction from membrane receptors like Tyr kinases, G protein-coupled receptors, and ion channels to the nucleus and participates in various cellular processes like cell proliferation, differentiation, and survival [82,83]. Various studies have reported that *ERK1/2*-*RUNX2* signaling enhances the osteogenic response of MSCs [84,85]. The activation of the ERK signaling pathway via the suppression of SPRY4, a known inhibitor of the MAPK/ERK signaling pathway, significantly enhanced the osteogenic differentiation of ADSCs, as indicated by the upregulation of key osteogenic markers such as ALP and OPN and increased levels of calcium deposition [86]. In vivo studies using a calvarial defect BALB/c nude mouse model further demonstrated that SPRY4 suppression leads to higher bone volume and trabecular number, suggesting that targeting SPRY4 could be a promising strategy for enhancing bone regeneration through the activation of the ERK1/2 signaling pathway [86]. Furthermore, another study identified the ERK1/2 signaling pathway as a key mechanism in the process of using deproteinized antler cancellous bone, a scaffold closely resembling natural bone in its composition and structure, to enhance the osteogenic potential of ADSCs [87]. As a result, seeding ADSCs into cancellous bone promoted the activation of *ERK1/2* and *RUNX2* in a rabbit mandibular bone defect model, suggesting that *ERK1/2* signaling plays a critical role in the improvement in bone regeneration [87].

### 4.6. PDGF-BB Signaling

PDGF-BB, a member of the platelet-derived growth factor (PDGF) family proteins that are composed of five isoforms, has gained significant attention due to its promising potential in tissue repair like bone regeneration and wound healing [88,89,90,91]. The osteogenic responses to PDGF-BB in ADSCs and BMSCs were compared and investigated, indicating that BMSCs do not enhance mineralization in response to PDGF-BB, while ADSCs exhibit significantly increased mineralization and the upregulation of osteogenic genes such as *RUNX2* and *OCN* [92]. This effect was further confirmed through in vivo experiments, where ADSCs overexpressing *PDGF-BB* improved bone regeneration in a murine calvarial defect model, highlighting the importance of targeting *PDGF-BB* signaling in ADSCs as an effective approach for bone tissue engineering compared to BMSCs [92].

### 4.7. Interplay of Multiple Signaling Pathways

It is widely accepted that the synergistic interplay of multiple pathways, such as BMP, ERK, fibroblast growth factor (FGF), Focal Adhesion Kinase (FAK), and Wnt/β-catenin signaling, orchestrates the complex process of ADSC osteogenesis. The role of Twist-related protein 1 (TWIST1), a transcription factor involved in cell lineage determination and differentiation, in the regulation of the osteogenic induction of human ADSCs was evaluated [93,94]. The silencing of TWIST1 enhanced the osteogenic potential of ADSCs in vitro, which led to the activation of the BMP and ERK/FGF signaling pathways and in turn upregulated the transcriptional coactivator PDZ-binding motif (TAZ), a key factor in osteoblast differentiation [94,95]. Additionally, the introduction of short hairpin TWIST1-treated ADSCs successfully rescued the calvarial defects in mice 10 weeks after surgery. The inhibition of these pathways or co-silencing of TAZ reversed the osteogenic effects, further highlighting the importance of the cooperation of TWIST1, BMP, TAZ, and ERK/FGF signaling to enhance bone regeneration in clinical applications [94]. Furthermore, another study reported the synergistic effects of FAK and BMP9 on the osteogenic differentiation and bone formation of ADSCs [96]. As a result, BMP9 enhanced FAK phosphorylation, leading to the increased expression of osteogenic markers such as *RUNX2* and enhanced ALP activity. Knocking down FAK significantly reduced the osteogenic potential of ADSCs, suggesting that FAK is crucial for BMP9-induced osteogenesis [96]. The study further revealed that FAK and BMP9 promote osteogenesis through the Wnt/β-catenin signaling pathway, rather than the Smad pathway [96]. These findings highlight the importance of the FAK-BMP9-Wnt/β-catenin signaling cascade in enhancing bone regeneration.

## 5. Enhancement of the Osteogenic Potential of ADSCs via Genetic Modification

*BMPs* are multi-functional growth factors that are members of the TGF-β family [97]. Among the 20 BMP family members that have been discovered and characterized [98], *BMP2* demonstrates the most potent ability to stimulate bone formation in vivo, making it a critical target for studies in osteogenesis and regenerative medicine [99,100,101]. To further improve the efficiency of the osteogenic differentiation of ADSCs and subsequent bone formation, the combination of genetic modification of *BMP2* and other factors is often applied. 

*RUNX2* is a crucial transcription factor for osteoblast differentiation [102,103]. Lee et al. transfected ADSCs with the *BMP2/RUNX2* expression vectors and then assessed the osteogenic potential both in vitro and in vivo [104]. As a result, the co-delivery of exogenous *BMP2* and *RUNX2* significantly increased the ALP activity, expression of osteogenic markers, and calcium deposition in vitro. In vivo studies further revealed that the ADSCs overexpressing *BMP2* and *RUNX2* exhibit substantially more bone formation compared to those overexpressing *BMP2* alone, highlighting the effectiveness of the co-delivery of these two genes for bone regeneration [104].

Growing evidence has indicated that microRNAs (miRNAs) are involved in the regulation of osteogenic differentiation [105]. In particular, miR-148b has been reported to enhance the osteogenesis of human ADSCs and rat BMSCs [106,107]. By utilizing a miRNA-expressing baculovirus vector system, ADSCs were engineered to co-express *BMP2* and miR-148b [108]. As a result, transfected ADSCs exhibited significantly improved osteogenesis compared to controls. In vivo studies further revealed that the transplantation of modified ADSCs results in the formation of bone that fills up to 94% of the defect area and 89% of the defect volume within 12 weeks, after seeding into critical-sized calvarial defects in nude mice [108]. Further research has elucidated the underlying mechanism, revealing that miR-148b directly targets the *NOG* gene, and the product of this gene, Noggin, functions as an antagonist to BMPs negatively regulating BMP-induced osteogenic differentiation and bone formation [109]. These findings suggest that the modulation of *BMP2* and microRNA can synergistically enhance bone regeneration and provide a promising strategy for treating large calvarial defects.

CRISPR interference (CRISPRi) uses a catalytically inactive Cas9 protein and single-guide RNA to repress sequence-specific genes, which is a programmable, highly efficient, and specific method for gene silencing [110]. The utilization of CRISPRi to directly target *NOG* provides an alternative method to enhance ADSC-mediated bone regeneration. A hybrid baculovirus system was developed to co-deliver *BMP2* and the CRISPRi machinery into ADSCs [111]. As a result, the CRISPRi-mediated knockdown of *NOG*, alongside *BMP2* overexpression, significantly improved the osteogenic differentiation of ADSCs and enhanced calvarial bone healing after seeding into rats, as evidenced by significantly increased bone area, bone volume, bone density, and matrix mineralization compared to that of the control group, highlighting the potential of coupling the CRISPRi strategy with the overexpression of *BMP2* to further enhance the osteoinductive potential of ADSCs in bone tissue engineering [111].

## 6. Enhancement of the Osteogenic Potential of ADSCs via miRNAs

miRNAs are small non-coding RNA molecules, typically 21–25 nucleotides long, which play a crucial role in regulating gene expression at the post-transcriptional level [112,113]. The interaction of miRNA with the 3′ untranslated region of target mRNA typically results in mRNA degradation and the inhibition of translation. The specific outcomes can vary based on the interaction with other sites and cellular contexts [114]. It is well studied that miRNAs can significantly enhance the osteogenic differentiation of ADSCs through the regulation of various osteogenic signaling pathways like TGF-β, BMP, and Wnt signaling and by targeting osteogenic inhibitors [115,116,117,118,119,120,121].

By using microarray data, six key miRNAs (miR-143-3p, miR-135a-5p, miR-31-5p, miR-22-3p, miR-193b-3p, and let-7i-5p) were identified to play a critical role in the osteogenic differentiation of human ADSCs [122]. Additionally, dihydropyrimidinase-like 3 was predicted to be the hub gene regulated by most miRNAs. Functional analysis also showed that the silencing of dihydropyrimidinase-like 3 promotes osteogenic differentiation, while its overexpression inhibits this process, highlighting the potential of targeting a specific miRNA-mRNA network to enhance bone regeneration and treat craniofacial bone defects [122].

miR-214 has been reported as a suppressor of bone formation through its direct targeting of activating transcription factor 4, a key regulator of osteoblast function [123]. A novel Cre/loxP-based hybrid baculovirus vector was introduced to knock down miR-214 in ADSCs, which led to the activation of Wnt signaling and the increased expression of β-catenin and *RUNX2* [124]. Additionally, engineered ADSCs successfully promoted osteogenesis and enhanced the repair of critical-sized bone defects in a rat model, suggesting the potential of inhibiting miR-214 for bone regeneration [124].

miR-146a is well studied as a dominant and negative regulator of the innate immune response and plays an important role in immune system homeostasis [125,126]. Additionally, the inhibition of miR-146a expression is associated with the enhanced megakaryocytic differentiation of hematopoietic progenitor cells, revealing the impacts exerted by miR-146a on cell differentiation [127,128]. miR-146a was identified as the most significantly downregulated miRNA during the *BMP2*-induced osteogenesis of Sprague Dawley rat ADSCs, indicating that miR-146a is a negative regulator during the process [115]. Furthermore, the overexpression of miR-146a significantly inhibited osteogenic markers such as *RUNX2*, Osterix (*OSX*), and *OPN* in ADSCs, while the repression of miR-146a enhanced their expression and promoted osteogenesis [115]. Moreover, miR-146a directly bound and inhibited the expression of SMAD4, a key coactivator in the *BMP2* signaling pathway, thereby reducing the activation of downstream osteogenic genes [115]. Most importantly, the inhibition of miR-146a in ADSCs and the subsequent transplantation of engineered ADSCs to treat the rat models with critical-sized cranial defects showed significantly improved bone regeneration compared to controls, as evidenced by higher bone volume fraction, bone mineral density, and trabecular number in the newly formed bone tissue [115]. These findings indicate that the repression of miR-146a in ADSCs may provide an alternative approach to stimulate craniofacial bone reconstruction.

Exosomes are extracellular vesicles produced by all cells and act as natural carriers to deliver numerous cargos including miRNAs between different cells for communication [129]. The miRNA expression profiles of exosomes derived from undifferentiated and osteogenically differentiated human ADSCs were compared and investigated, which identified 201 upregulated and 33 downregulated miRNAs in the differentiated group, and only the exosomal miRNAs that are derived from osteogenically differentiated ADSCs can promote the osteogenic differentiation of other ADSCs [117]. Specifically, the miR-130a-3p/SIRT7/Wnt axis was shown to promote the osteogenic differentiation of ADSCs [117].

## 7. Enhancement of the Osteogenic Potential of ADSCs via Scaffolds

For the application of ADSCs in craniofacial bone regeneration, scaffolds are generally used to provide a three-dimensional structure that mimics the natural extracellular matrix of bone tissue. This structure is crucial for supporting the viability, proliferation, and differentiation of ADSCs into osteoblasts and for guiding new bone formation [130,131,132,133]. In addition, well-designed scaffolds can degrade at a rate that matches new bone formation, allowing for the gradual replacement of the scaffold with newly formed bone tissue [134]. Three-dimensionally printed scaffolds can be tailored to fit the specific shape and size of craniofacial bone defects, ensuring better anatomical fit and functional outcomes [135,136].

The combination of polycaprolactone (PCL) and gelatin (Gel) nanofibers provides the advantages of high mechanical strength and improved biodegradation properties for bone scaffolds [137,138,139]. In addition, nanohydroxyapatite (nHA) and vitamin D3 (Vit D3) are known for their osteoconductive potential and stimulation of bone formation and mineralization [140,141]. PCL/Gel/nHA/Vit D3 composite scaffolds were developed using electrospinning and tested for their ability to promote bone tissue formation [142]. The results showed that the composite scaffolds significantly enhance the osteogenic activity of ADSCs, leading to increased ALP activity, greater mineralization, and the upregulated expression of key osteogenic markers like *Col-I*, *BGLAP*, and *RUNX2* [142]. These findings suggest that seeding ADSCs into PCL/Gel/nHA/Vit D3 composite scaffolds offers a potential method to improve bone regeneration.

Nanofibrillar cellulose (NFC) hydrogel is a non-cytotoxic, plant-derived material that is composed of cellulose nanofibrils and has various biomedical applications [143]. In addition, electrical stimulation (ES) has been reported to promote the osteogenic induction of human mesenchymal stromal cells via the activation of *BMP2* [144]. NFC hydrogel, when combined with ES, significantly increased the osteogenic differentiation of ADSCs, as evidenced by the elevated ALP activity, enhanced calcium deposition, and upregulated expression of osteogenic markers like *OCN* and *OPN* [145].

*Col-I* is a crucial component that acts as a structural protein in the extracellular matrix of bone tissue [146]. Furthermore, β-tricalcium phosphate (β-TCP) serves as an effective bone substitute due to its biocompatibility, osteoconductivity, and biostability [147]. To improve the treatment of critical bone defects, a novel composite material combining ADSCs with a *β-TCP/Col-I* fiber scaffold was developed [148]. The composite scaffold apparently facilitated ADSC attachment and proliferation with no observed cytotoxicity and significantly enhanced the expression of osteogenic markers like *ALP*, *OCN*, and *OPN* and calcium deposition as compared to the control group [148]. 

Central necrosis within cell aggregates remains a challenge for the application of scaffolds [149,150]. To overcome this problem, a novel cell transplantation platform called CellSaic has been developed, which uses micropieces of a recombinant peptide like the alpha-1 sequence of human *Col-I* to create a mosaic structure [151]. CellSaic provides an optimized microenvironment to enhance the osteogenic differentiation of MSCs and promotes the repair of rat mandibular congenital defects [152]. For the clinical transplantation of cells, xenogeneic products such as fetal bovine serum are another concern due to the possibility of carrying pathogens and viruses and should be avoided [153]. Sun et al. evaluated the osteogenic potential of rat ADSCs cultured in a xeno-free environment compared to those cultured with fetal bovine serum [154]. The xeno-free ADSCs showed enhanced osteogenic differentiation, as evidenced by increased calcium deposition, ALP activity, and upregulated osteogenic gene expression as compared to control group. The transplantation of xeno-free ADSCs into CellSaic further demonstrated superior bone regeneration in the rat model with mandibular bone defects, suggesting that using xeno-free culture conditions in combination with CellSaic constructs could improve the safety and efficacy of ADSCs-based craniofacial bone regeneration [154].

Polypropylene carbonate (PPC) is a biodegradable polymer known for its ability to degrade into non-toxic byproducts including water and carbon dioxide and has been widely used in industry [155,156]. In the field of bone engineering, the modification of the surface roughness and hydrophilicity of PPC significantly enhanced the osteogenic differentiation of rat BMSCs [157]. Additionally, silicon nitride (SiN) is recognized for its excellent mechanical properties and osteogenic potential [158]. Recently, the osteogenic differentiation of ADSCs in a composite material of PPC and SiN was investigated [159]. The study showed that a 20% SiN content (PSN20) in the composite significantly enhanced the osteogenic differentiation of ADSCs and improved bone formation in vivo, highlighting the potential of application of ADSCs and the PPC/PSN20 composite for treating bone defects in tissue engineering [159].

## 8. Enhancing Osteogenic Potential of ADSCs via Chemical, Biological, and Physical Factors

To further strengthen the osteogenic potential of ADSCs, other strategies like using chemical factors, hormones, growth factors, and the modification of the physical properties of scaffolds are often applied for bone regeneration. 

Curculigoside is phenol that can be isolated from various plants such as the rhizome of Curculigo orchioides Gaertn [160]. This compound significantly promoted the osteogenic differentiation of ADSCs via the activation of the PI3K/Akt signaling pathway [161]. In addition, ascorbate-2-phosphate is a vitamin C derivative that is often supplemented in osteogenic induction culture medium to stimulate collagen formation [162]. The treatment of ADSCs with ascorbate-2-phosphate led to the formation of ADSC sheets that exhibited higher ALP activity and earlier mineralization as compared to separate ADSCs, indicating a stronger and more sustained potential for bone formation [163]. TAZ is a transcriptional coactivator that plays a crucial role in the osteogenic differentiation of rat BMSCs through the activation of the PI3K/AKT signaling pathway [164]. The regulation of TAZ in the osteogenesis of human ADSCs has also been investigated by using a pharmacological activator, TM-25659, to stimulate TAZ in ADSCs [165]. As a result, the activation of TAZ significantly increased the osteogenic potential of ADSCs both in vitro and in vivo, which was achieved by enhancing the interaction between TAZ and *RUNX2* and the increased expression of osteogenic markers like *OCN* [165]. 

The parathyroid hormone plays an important role in protecting *RUNX2* expression and promoting osteoblast proliferation and differentiation and is the only FDA-approved agent to treat osteoporosis [166,167,168]. An et al. reported that the parathyroid hormone (1–34) enhances the phosphorylation of Salt-Inducible Kinase 2, which in turn upregulates Wnt4 expression in osteoinduced ADSCs [169]. This signaling cascade promotes bone formation by increasing the expression of osteogenic markers such as *RUNX2*, *OSX*, and *OCN*, suggesting that targeting these pathways could improve the effectiveness of ADSC-based bone regeneration therapies [169].

FGF2 has been reported to stimulate osteoblast differentiation through the regulation of the Wnt signaling pathway [170]. Additionally, another study indicated that hepatocyte growth factor (HGF) promotes the osteogenic capacity of human BMSCs. Moreover, ADSCs from elderly or diseased individuals were found to exhibit reduced osteogenic potential due to lower levels of critical growth factors including FGF2 and HGF, showing a close correlation of ADSC osteogenesis with the expression of these growth factors [171]. Indeed, combinatory treatment with FGF2 and HGF significantly improved the osteogenic differentiation of ADSCs from elderly donors, as evidenced by the increased expression of *RUNX2*, *OSX*, and *ALP* and greater calcium deposition compared to non-primed controls, as well as the elevated secretion of *BMP2* and vascular endothelial growth factor that are crucial for bone formation [171]. Most importantly, the transplantation of primed ADSCs enhanced bone-forming capacity in vivo, as revealed by well-formed bone structures and the significant expression of *OCN* [171].

Physical factors such as the wettability and roughness of scaffold material often affect the efficacy of the osteogenic differentiation of osteoblasts and human BMSCs [172,173]. Stepanovska et al. determined the impact of various surface modifications of the titanium alloy scaffold Ti6Al4V on the growth and osteogenic differentiation of ADSCs [174]. They found that Ti6Al4V with a surface roughness of Ra 60–70 nm, after the treatment of brushing or anodizing, exhibited the highest osteogenic differentiation and satisfactory growth levels, as revealed by the increased expression of *RUNX2*, *Col-I*, and *OPN*, elevated calcium consumption by the cells, and enhanced ALP activity [174]. This study highlights the importance of the modification of scaffold surface roughness to enhance the osteogenic capacity of seeded ADSCs for bone regeneration.

## 9. Clinical Reports of ADSCs Facilitating the Repair of Craniofacial Bone Defects

Autologous bone grafting remains the gold standard for craniofacial bone regeneration for adults [175]. However, in the case of large bone defects occurring in children, the repair would be difficult due to the limited amount of autologous bone available and the continuous growth of children. In 2004, Lendeckel et al. described the first case report of the use of ADSCs to facilitate the repair of widespread traumatic calvarial defects in a 7-year-old girl after a fall [176]. The girl initially underwent a bilateral decompressive craniectomy due to the refractory intracranial hypertension after the accident. However, calvarial bone resorption and chronic infection occurred after the subsequent replantation and fixation of calvarial fragments. Therefore, the ADSCs harvested from the patient’s autologous gluteal region fat depot, milled cancellous bone grafts, and autologous fibrin glue scaffold were applied to the calvarial defect sites to promote the craniofacial bone regeneration process. Three months after operations, CT scans showed improved symmetrical calvaria contour and ossification at the site of trauma, indicating uneventful postoperative healing [176]. Notably, although good prognosis was observed, the beneficial effects in the repair of calvarial defects that are exerted solely by ADSCs remain to be determined due to the cell population heterogeneity arising from harvest procedures that were described in the case report and the combination of cancellous bone graft therapy during the treatment.

Sandor et al. reviewed the use of ADSCs to reconstruct cranio-maxillofacial hard-tissue defects in 13 patients [177]. The ADSCs were harvested and isolated from patient autologous adipose tissue located in the anterior abdominal wall and then seeded onto resorbable scaffolds, including bioactive glass and β-TCP, with or without the addition of *BMP2*. These constructs were then implanted to treat defects in areas such as the frontal sinus, cranial bone, mandible, and nasal septum. In this study, ten out of thirteen cases showed successful integration, with bone resorption observed in two cranial defect cases where nonrigid resorbable meshes were used. One case of nasal septum reconstruction failed due to patient noncompliance [177]. This study highlights the potential of ADSCs in cranio-maxillofacial reconstruction, while it also notes the need for careful patient selection and scaffold choice.

The repair of large calvarial defects remains a challenge due to the restrictions of autologous bone grafts and alloplastic materials. Thesleff et al. developed a novel method for cranial reconstruction by using a combination of ADSCs and β-TCP granules [178]. The ADSCs were harvested from subcutaneous abdominal fat from the patients with large calvarial defects, expanded, and seeded onto β-TCP granules, then implanted into the cranial defects. Surprisingly, the results showed successful bone regeneration, as indicated by increased Hounsfield units on CT scans, which approached normal bone density over time [178]. However, their six-year long-term follow-up results revealed that three of the five patients required re-operation due to graft-related issues, including graft resorption and late infection [179]. Further research is needed to optimize the technique before broader clinical application.

Khojasteh et al. described two case reports that examined the application of buccal fat pad-derived stem cells (BFPSCs) combined with guided bone regeneration (GBR) for the reconstruction of large alveolar bone defects following the extraction of multiple impacted teeth [180]. The first case was a 19-year-old woman with cleidocranial dysplasia, who had 11 impacted teeth in the upper jaw and 13 in the lower jaw. After the teeth were surgically removed, the large bone defects created were filled with BFPSCs loaded onto natural bovine bone mineral (NBBM) granules. Six months post operation, dental implants were successfully placed in both the maxilla and mandible. Radiographic evaluation at 10 months revealed thorough bone regeneration and implant survival. The second case involved a 22-year-old man with a similar condition, who had 12 impacted teeth in the upper jaw and 10 in the lower jaw. The same procedure was performed, and after 12 months, the patient received dental implants. Follow-up at 48 months showed complete bone regeneration and stable implant integration [180]. Both cases demonstrate that BFPSCs, when used in conjunction with NBBM and GBR techniques, can effectively promote significant three-dimensional bone formation, facilitating the successful placement and survival of dental implants in areas with large alveolar bone defects.

## 10. Conclusions and Perspectives

Substantial advancements have been achieved in preclinical research that aims at enhancing the osteogenic potential of ADSCs for craniofacial bone regeneration. These developments encompass a wide array of innovative strategies with each targeting different aspects of ADSC biology and function, including (1) the selection of specific ADSC subpopulations with strengthened osteogenic capacity; (2) the intervention of key signaling pathways; (3) the genetic modification of crucial transcription factors for osteoblast differentiation; (4) the regulation of miRNAs to fine-tune ADSC behavior at the post-transcriptional level; (5) the design of innovative biomaterials and scaffolds to provide optimal three-dimensional environments for ADSC growth and differentiation; and (6) the involvement of various chemical, biological, and physical factors to further stimulate the osteogenic differentiation potential of ADSCs (Figure 1). However, based on our literature research, we identified only four clinical case reports that described the use of ADSCs and outcomes for craniofacial bone regeneration [176,178,179,180]. Furthermore, there is currently no Food and Drug Administration-approved ADSC product specifically for bone regeneration [181], suggesting the existence of a gap between the promising results observed in preclinical studies and the actual clinical application of ADSCs. Notably, the limitation is mainly due to the rigorous controls imposed by national regulatory frameworks, based on the consideration of the safety and efficacy of ADSC therapy [182]. To overcome the gap, it is important to develop more consistent protocols for the isolation and expansion of specific subpopulations of autologous ADSCs with enhanced osteogenic capacity, carefully select proper scaffold materials and other osteogenic factors, and cautiously evaluate the outcomes or adverse effects after implantation into craniofacial bone defect regions before the final translation into clinical products.

## Data Availability

Not applicable.
